# Symptom Control Trials in Patients With Advanced Cancer: A Qualitative Study

**DOI:** 10.1016/j.jpainsymman.2015.05.009

**Published:** 2015-11

**Authors:** Tom Middlemiss, Mari Lloyd-Williams, Barry J. Laird, Marie T. Fallon

**Affiliations:** a Edinburgh Cancer Research Centre, University of Edinburgh, Western General Hospital, Edinburgh, United Kingdom; b Academic Palliative and Supportive Care Studies Group, University of Liverpool, Liverpool, United Kingdom

**Keywords:** Palliative care, research, cancer

## Abstract

**Context:**

Symptom control research in patients with advanced cancer is not common. This may be the result of a belief that this research is unethical, not practical, or that patients are not interested. However, the experiences of cancer patients who have actually taken part in symptom control research near the end of life have never been detailed.

**Objectives:**

The objective was to explore the experiences of patients with advanced cancer who had taken part in symptom control trials.

**Methods:**

A prospective two-center study was undertaken using grounded theory methodology. Theoretical sampling was used to recruit patients from one of two double-blind, randomized, placebo-controlled trials studying novel analgesic agents for cancer-related pain. Participants completed one semistructured interview. Recruitment and interviewing continued until data saturation was achieved.

**Results:**

Twenty-one participants were recruited. Fifteen (71%) were male, with a mean age of 62 years. Key themes identified included reasons for trial participation, participants' interactions with the trial staff, and participants' responses to the effect the trial had on their pain. In general, participants regarded taking part in a clinical trial as a positive experience, and potentially improving overall well-being. Crucially, this was not related to whether there had been an improvement in symptoms.

**Conclusion:**

The findings provide grounds for optimism that patients with advanced cancer may benefit from taking part in symptom control trials, supporting the paradigm that participation in symptom control research should be encouraged in this population.

## Introduction

Patients with an advanced life-limiting illness such as cancer describe multiple symptoms including pain, fatigue, nausea, and breathlessness.^1^ The fact that incidence and prevalence levels remain high indicates that these symptoms are not always optimally managed. One of the main reasons for this is the lack of an evidence base for the treatment of symptoms.

Conducting research in these patients has traditionally been regarded as challenging because of ethical, institutional, philosophical, and practical reasons. As a consequence, there remains a paucity of evidence for treatment of common symptoms. There is a clear need to improve the care of patients with life-limiting illness through robust, meaningful, and appropriate research.^2^, ^3^

There is an intrinsic resistance from health professionals, including those in palliative care, to encourage patients to participate in palliative care research, especially clinical trials, with paternalistic assumptions regarding vulnerability and cause of undue patient distress as common themes.^4^ The concept of a placebo arm can be of particular concern to many health professionals when “standard” treatments are available, even though this argument may be flawed when considering that the evidence for comparative therapies may be based on anecdotal evidence alone.^5^ Additionally, research proposals may be met with resistance from ethics committees and health care practitioners alike, with documented issues including concerns regarding data protection, unsolicited contacting of patients, choice of terminology to prevent distress to patients, a view that dying patients should not be troubled by research, poor understanding of methods used in palliative care research, and a desire to prevent “unethical research.”^6^, ^7^, ^8^, ^9^

Conversely, previous studies have suggested that patients with advanced cancer want to participate in palliative care research. However, these studies asked patients about their attitudes to hypothetical research studies.^10^, ^11^ Very little is known about the experiences of patients who have *actually* participated in symptom control trials. The aim of this novel piece of research was to examine the experiences and attitudes of patients with advanced cancer who took part in symptom control trials.

## Methods

A prospective, two-center, qualitative study was undertaken using grounded theory methodology.^12^, ^13^, ^14^ Ethical approval was obtained for this study (West of Scotland Ethics Service, ref 09/s0703/104). Patients were recruited from two regional cancer centers in Scotland serving a population of approximately three million people. Eligible patients met the following criteria: a diagnosis of advanced cancer (defined as metastatic and/or incurable), aged 18 years or older, able to give written informed consent, and have participated previously in one of two symptom control trials. The symptom control trials were both double-blind, placebo-controlled clinical trials studying ketamine for neuropathic pain and pregabalin for bone pain as novel analgesic agents (clinical trials: NCT01316744 and NCT00468845; see Appendix I for further details of the clinical trials). Exclusion criteria were under 18 years of age, a cancer diagnosis with a potentially curative outcome, and patients in the dying phase of their illness. The choice of this final exclusion criteria is discussed further on.

It is important to note that defining a palliative care population is difficult as it is based on needs, not diagnosis nor stage of disease.^15^ Attempts have been made to address this challenge.^16^ To study such a heterogeneous group would pose greater logistical challenges than could be surmounted in one study. The decision was made to study only patients with an advanced cancer diagnosis. Therefore, when the term palliative population is used herein, this refers to patients with a diagnosis of advanced, metastatic, incurable cancer.

### Patient Sampling

The researcher/interviewer (T. M.) had no involvement with the original clinical trials. The details of all patients completing trial participation were made available to him by trial staff. If patients were thought to be suitable for interview, they were contacted either by T. M. directly or by the research team to enquire about their desire to take part in this study. Theoretical sampling was used to select patients as the interviews proceeded. Patients were chosen to provide the maximum diversity of experiences from the trials.^17^ Initially this sampling related to age, sex, trial, and geographic location. Geographic location was important as there were some different members of the trial staff at the two recruitment sites. As the study progressed, the sampling was influenced by participants' responses. Subsequent strategies involved approaching patients with variable pain responses to the trial (using pretrial and posttrial validated pain scores that had been used in the trials), patients who had contacted the researchers after the trial period had ended, and those patients who had withdrawn from the trials or who had been withdrawn from the trials early for any reason. It was felt that this final group of patients possibly would be negative cases that may give an alternative view of their experiences of being on the trial. All these patients were contacted; however, all declined to take part in this study. In comparison to other palliative care research, patients enrolled in the studied trials were largely still living independently at home with an Eastern Cooperative Oncology Group score of 1–2. Therefore, functional status was not chosen as a sampling variable.

### Interview/Analysis Process

A semistructured interview using open questions was carried out.^18^, ^19^ Participants may have completed the trial, withdrawn from the trial, or been withdrawn from the trial. Every effort was made to interview participants within two weeks of completing their trial involvement. In some cases, the time between completing the trial and interview was longer, for example, at the start of the interview process when patients had completed the trial longer than two weeks previously. The interviews were recorded, transcribed, and a verbatim copy of the transcript was sent to the participant, inviting him/her to comment or elaborate on any points discussed.^20^ The intention of this was to allow patients to elaborate or clarify any themes that they had discussed and increase the rigor of the study. However, no patients took up the opportunity to contact the researchers for further discussion, although some did comment that they had enjoyed having a transcript of the interview.

Participants were given the choice of the interview taking place in their home or in a hospital/research setting. It was requested that participants be interviewed alone; however, some participants did want to have someone else present, and this request was granted. Without trying to create a barrier between interviewer and participant, all efforts were made to ask that the participant answer all the questions, and any comments made by anyone else were not analyzed.

In keeping with a grounded theory approach, data analysis took place concurrently with the data generating process of interviewing.^14^ Known as constant comparison, analysis of preliminary data influenced subsequent participant selection and question generation, which in turn prompted further analysis of all generated data. (Copies of the initial and final question guide are listed in Appendix II.) T. M. undertook line-by-line coding of the transcripts and initial data analysis. This analysis and emerging themes were then discussed with B. J. L. Analysis of the generated data developed core categories and a central theory that described the studied phenomenon. A clear audit trail of this process was kept through memo writing and field notes. Patients were recruited and interviewed until a suitable degree of data saturation was reached; in other words, until it was felt that no additional new data were likely to be generated from further interviews.^21^

Constructivist grounded theory puts the role of the researcher and their background at the forefront of the research and its interpretation. To put the researcher into the context of the research, T. M. is a physician training to specialize in palliative medicine. As this was not concealed from participants, this may have had an impact on how participants viewed T. M. (e.g., as part of the trial staff rather than an independent researcher), the manner and content of the responses they gave him and whether they were fully able to share all their views. T. M.'s background and status in the eyes of participants are acknowledged factors that will have had an impact on the research findings.

## Results

**Fig. 1:**
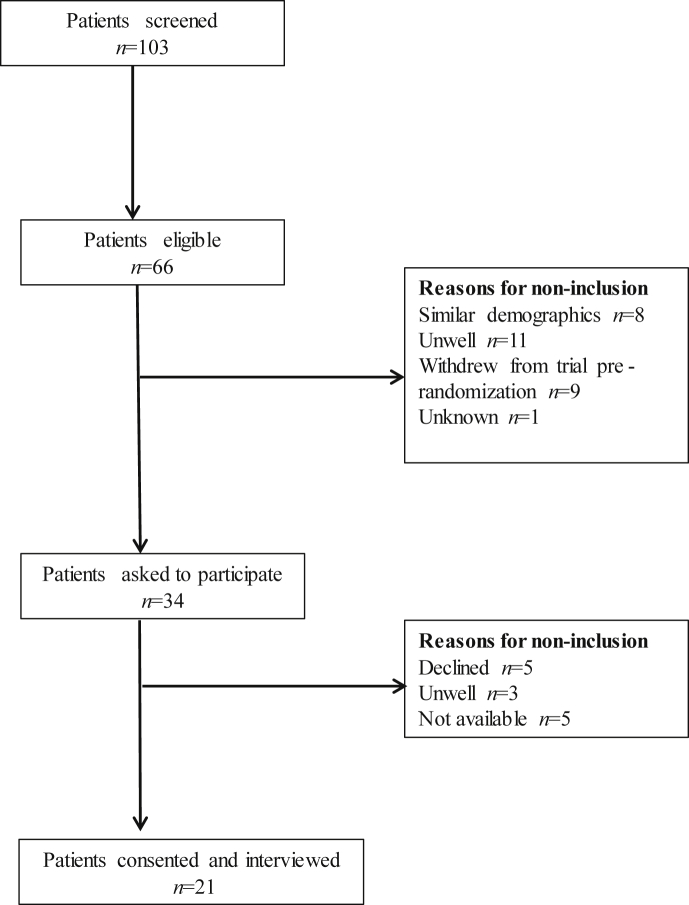
This CONSORT diagram shows the patients who were recruited into this study. It illustrates the total number of patients who were eligible for the study and the reasons why some were not contacted at different stages of the recruitment process.

The screening and recruitment process are shown in Fig. 1. During the period of study recruitment, 103 patients completed both trials. Of this number, 66 patients were eligible to be interviewed, 34 patients were contacted, and 21 were interviewed. Reasons that patients were not contacted included those who were too similar in sampling profile to those previously interviewed, patients withdrawing from the trial before taking any medication, and those who were too unwell or dying to be felt appropriate to be contacted. Of the patients that were contacted but not interviewed, reasons included patient rejection, patients not replying to the contact, and patients becoming too unwell between agreeing to take part and the interview date.

Participant demographics and disease characteristics are shown in Table 1. Most participants were male (*n* = 15, 71%), and the mean age was 62.1 years. The most common cancer type was prostate. Although the intention was not to produce a representative sample of eligible patients, comparison between those interviewed and those eligible shows similarities akin to representation.

The results are presented along the linear time frame of trial participation, namely participant experiences before starting the trial; participant experiences during the trial; and patient reflections on being involved in a trial. However, the themes that are described are those that arose from participant responses and the subsequent data analysis. The central theory of well-being, which links all aspects of participants' trial participation, is also presented. For ease of comprehension, colloquial language has been amended.

### Participant Experiences Before Starting a Trial

**Fig. 2:**
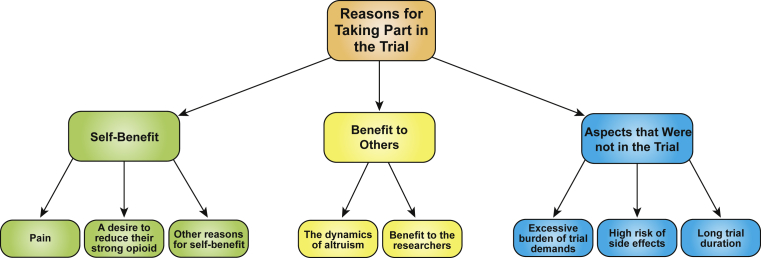
Reasons for trial participation. This figure illustrates the wide range of reasons why participants wanted to take part in the clinical trial. Participants might have several of the reasons outlined. The reasons were grouped into self-benefit, benefits to others, and aspects that were not in the trial.

The reasons for trial participation are shown in Fig. 2. The awareness of the concept of clinical trials varied among the patients studied. Some patients had never heard of clinical trials. Other patients had spoken to friends who had heard of clinical trials. Some patients had been on other clinical trials in the past or had known friends or relatives who had taken part in clinical trials. Through the patient information sheets and contact with the trial staff, participants subsequently felt well informed about their specific trial. When considering whether to agree to take part in a clinical trial, participants considered what the trial would involve for them and in particular weighed whether the trial would have a greater perceived benefit than burden.

Female, 58 years, breast carcinoma: Ah, yes, I spoke to my husband, my son, uh, my Dad (laughs), but eh, really it was a no brainer, it was eh, it was the idea of pain relief is always a brilliant thing and eh, um, I do agree with the principle of why they're looking at it.

Participants described positive reasons for taking part in the trial within the context of someone benefitting. This “someone” could be themselves, with motivations for taking part including hopes for reducing their pain, reducing the quantity of opioids they were taking, or achieving a better structure to their medications and care. However, someone else could also benefit. Participants cited examples such as, future patients or the researchers conducting the trials benefitting from their participation. In the eyes of the participant, the benefit from the trial did not have to be solely for them.

Male, 48 years, pancreatic carcinoma: At the end of the day, if you didn't have people to try trials … I wouldn't be at this stage with my pain, so you've got to, there has got to be guinea pigs along the line somewhere.

Participants described three aspects of the trial design that appeared favorable when considering enrolling. These aspects were what the trials did not contain rather than what they did an apparent lack of excessive trial demands, a perceived lack of possible side effects, and the lack of a lengthy trial period.

Male, 52, prostate carcinoma: I was persuaded a bit more by the fact that it was only going to last for a short time and it wasn't going to really, cause me any hassle, in the sense of having to go anywhere, or go to the hospital, get ah … it was all going to be done locally or on the end of a telephone.

When questioned about their view on being part of a trial containing a placebo, participants had a good understanding of the concept of placebo. Some participants considered a placebo to be the central component of their trial and did not mind the possibility that they may receive one.

T. M.: What did you think about the fact that you might be on a placebo?

Female, 60, breast carcinoma: Well, not much, you know, somebody's got to be. It's the only way they can find out.

Some participants, however, showed a poor understanding of the concept of placebo by thinking that the drugs they received throughout their own trial could vary.

Male, 68, prostate carcinoma: Aye, some were, some must have been real and some of them were dummy, as far as, because the pain was not, I was not getting great pain, know what I mean, some time.

This statement illustrates that despite the interaction with the research staff and information sheets, there is still the possibility of misunderstanding regarding how or when the placebo is administered to patients.

### Participant Experiences During a Trial

The experiences of participants during the trial period were mostly positive; “fascinating” as one participant described it. One participant, who wanted a better structure to his care, felt he had received just that. Some participants described a very straightforward experience, largely devoid of positive or negative incident.

Male, 72, prostate carcinoma: It was just all, get the tablets, take the tablets, or hand them back or whatever and speak to the, speak to the girls. No there was nothing out of the ordinary.

The main themes that participants described were the impact of the trial on their pain and the interaction with the trial staff.

#### Impact of the Trial on a Participant's Pain

Some participants described dying as a preferable option to continuing to suffer their pretrial pain. Therefore, the “relief beyond relief” at a good pain response was clearly described. Some were stoical about a failure to reduce their pain, whereas others described an understandable disappointment. However, this disappointment was not without the caveat that they knew what the outcome might be when enrolling in the trial.

T. M.: What did you think after you'd started the trial drug and there was no benefit?

Female, 58, breast carcinoma: I felt okay. I, I was not disappointed or anything because I thought well I'm doing it to help, it's a drug and it's a trial thing and you're helping other people and it did, it didn't bother me. Well, I would've been over the moon obviously had it helped.

#### Interaction with the Trial Staff

This was a continual and key theme of the study. Some participants held the trial staff in high regard, considering them “just like a friend.” Participants would describe calling trial doctors by their first names, trial nurses collecting prescriptions on their behalf, and delivering on promises. Participants felt that they were known personally by the trial staff. A relationship based on trust was built: “There's no ‘next please.’” There was a sense of security in being known and being able to contact a member of the trial team at any time. Conversely, other participants were more neutral, complimenting the competency of the trial staff without being effusive. This difference in perceived interactions with the trial staff was explored closely and is discussed later.

#### Negative Aspects of the Trial

No participants interviewed stated that they did not like being on the trials. A potential reason may be linked with the previously discussed fact of not being able to recruit any negative cases. However, some aspects of the trials were described in a negative way by some patients. Negative factors in a trial may lead to patient anxiety, which in turn may lead to attrition rate or a negative experience of trial participation. Negative factors that patients described related to some of the trial questions, side effects, the number of pills they were required to take, and talking with someone new.

Some participants found that trial-related questions regarding their pain were difficult to answer. For some, this was quantifying their pain on a numerical scale, whereas for others, it was choosing descriptive words for their pain. Although two participants interviewed had to withdraw from the trial because of side effects, in general, the reported side effects of hallucinations and somnolence were not described with anxiety or irritation because the participants found that their pain improved at the same time, balancing the impact of the side effects. Some participants noted that toward the end of a trial period, the quantity of tablets they had to take was enough to merit a comment when describing any negative features of the trial.

### Participant Thoughts About Being in a Trial

When participants reflected on taking part in these symptom control trials, it was often within the context of their pain. A participant who had a good pain response was also typically positive about the trial. For some participants, despite poor pain reduction and subsequent underlying feelings of disappointment, their overall experience of the trial was still positive. Factors relating to this were the alternative benefits that they took from the trial and the manner in which they viewed the failure of the trial to reduce their pain. Participants looked to other positives of the trial, such as altruism, when their pain did not decrease. They also viewed their participation in the trial within the context of the potential of receiving a placebo:

Male, 49, chronic myeloid leukemia: … that's part and parcel of these trials anyway. You know that before you go into the trial.

Participants were generally happy to have taken part in the trial. Some were happy to consider taking part in another trial again, whereas others reported that their body had had enough.

### Central Theory of Well-Being

Several questions permeated the interview findings and data analysis. Why were some participants so positive about aspects of the trial, whereas others were more neutral? Some participants were positive about the trial even without a reduction in their pain. What linked all the aspects of a participant's experience of a clinical trial?

The answer, and the central theory grounded in the data, lay in the impact on a participant's well-being. Well-being is a fluctuating equilibrium sensitive to internal and external influences and unique to each participant. Given the uniqueness of well-being, like pain, a comparison between individuals would be meaningless. The impact of the trial on each individual's well-being is unique. The affecting internal factors could be physical, emotional, or spiritual. The external factors could be the interaction with others or the loss of an activity, such as driving. When the participant described his reduction in pain as “relief beyond relief,” it was clear that this was having a positive impact on his well-being. The trial staff appeared to be willing to do things that boosted or maintained participants' well-being rather than to diminish it. Aspects of the trial, or care in general, could have a negative impact on a participant's well-being. Being faced with eight new tablets on top of the usual thirty tablets is one example (see Table 2 for examples of positive and negative aspects of the trial on a participants' well-being).

#### The Individuality of Well-Being

Influences from different aspects of the trial had different significance for different individuals. For example, the fluctuation in a participant's pain state could increase or decrease that participant's well-being by different amounts. Similarly, the benefit received from the input of the trial staff may be greater in some participants than others. When all the variable factors of the trial are taken into account, the overall outcome of the trial, whether positive or negative, is likely to vary between individuals. This might suggest why some participants found the trial to be a very positive experience, whereas for others, it was something that fitted into their daily routine without having much impact.

The fluctuation of an individual's well-being is dynamic. The researchers did not think of it as directly proportional to factors such as disease state or physical condition. Instead, these factors play a contributory role in the state of an individual's well-being. If the positive influences on an individual's well-being, perhaps emotional or spiritual, have a greater effect than the negative influences, potentially physical, it may explain why a participant who had no reduction in pain was willing to continue to take part in a clinical trial. It may be that an individual's personality will influence the positive factors that they take from a clinical trial. However, the total influence from the clinical trial on a patient's well-being is finite. Everything that goes on in an individual's life can increase or decrease their well-being. The influencing factors from a clinical trial could have a very small or very large impact on a person's overall well-being. The context of the trial in relation to an individual's overall well-being is beyond the scope of this study. What has been considered is the impact that being on a trial has on an individual's well-being.

## Discussion

This study provides valuable new information regarding patients with advanced cancer participating in symptom control trials—an area with a hitherto limited knowledge base. The trial process was found at least to be straightforward and even enjoyable for some patients. Although the principle motivation for patients to participate in the trials was to reduce their pain, altruism was a clear secondary reason. Lessons for the design of future symptom control trials can be drawn from the positive comments relating to the apparent simplicity and short duration of the trials, the minimal hospital involvement, and the high researcher contact.

The central theory of this study is that being in a clinical trial has the potential to improve patients' well-being, and this is echoed in the literature. Other research has focused on the improvement of physical symptoms to improve well-being.^22^ Although this sentiment cannot be argued with, this study has shown that even when physical symptoms do not improve, well-being can still be improved by participation in a clinical trial. The importance of the relationship between researcher and participant, which was found in this trial, has been recognized by others in the setting of advanced cancer. An improved “alliance” between patient and oncologist, as described by Trevino,^23^ had a positive impact simultaneously on patient's well-being and treatment adherence, reinforcing the argument that patients and researchers can both benefit from a positive impact on a patient's well-being through a clinical trial. Although dealing with life-prolonging treatment, rather than symptom management, Madsen^24^ found similarities in the clinical trial experiences of patients with advanced breast cancer, particularly relating to the relationship with the trial staff. “Positive psychological outcomes” are described in the hospice setting when describing health care professionals who invest in the patient relationship.^25^ Furthermore, a review of well-being in terminally ill patients included the themes of self-awareness, having satisfying relationships with and connections to others, and living with meaning—all of which could be achieved during trial participation.^26^

Excluding patients from study participation who were in the “dying phase of their illness” runs the risk of accusations of gatekeeping. The authors acknowledge this and consider gatekeeping to be a spectrum on which all must position themselves. Although the experiences of patients who had taken part in clinical trials were unknown, the researchers chose to take a stronger view towards deciding on a degree of gatekeeping. With the information discovered in this study pointing toward positive experiences from clinical trials and, by extrapolation, research in general, those patients who may be closer to dying could be considered for inclusion in studies such as the one described.

### Limitations

The present study has limitations. There is no typical palliative care patient because of variables such as performance status, diagnosis and prognosis. As such, no clinical trials in palliative care patients will cover this entire cohort. It is acknowledged that the participants interviewed in this study were of a higher performance status than a lot of palliative patients with further disease progression. In the same manner, the studied trials were not particularly arduous for the patients and this would have had some impact on the manner in which they viewed their particular trial and trials in general.

We endeavored to gather the widest ranges of participant experiences, consistent with the trial populations as a whole; however, we cannot ensure this was the case. In the knowledge that some patients withdrew themselves from the trials, and refused to participate in this study, some experiences of trial participation have not been fully explored. Additionally, we interviewed participants after they had finished a trial, and thus their opinions may be limited by recall bias. Nevertheless, we are confident that the findings provide a broad reflection of the range of the participants' experiences.

## Conclusion

This is the first study examining the experiences of patients with advanced cancer who have participated in symptom control trials. Although patients may derive an improvement in physical symptoms, trial participation alone can improve well-being, making the trial a *therapeutic opportunity*^27^ in and of itself. These findings suggest that conducting symptom control research in patients with advanced cancer is appropriate and may be beneficial, regardless of the effect of the trial intervention. Although these findings provide grounds for optimism, we welcome further assessment and validation of our work. We propose that future work in this area should focus on research for symptoms other than pain. Clearly, if our findings are supported, this would have considerable implications for symptom control research in patients with life-limiting illness, challenging the paradigm that conducting research in patients with advanced cancer is inappropriate.

## Figures and Tables

**Table 1:** Comparison of Recruited and Eligible Patients' Demographics

Demographics	Interviewed Patients *n* = 21	Eligible Patients *n* = 66
Gender		
Male	15 (71%)	38 (58%)
Female	6 (29%)	28 (42%)
Age, yrs		
mean	62.1	62.1
range	48–24	32–84
Diagnosis		
Breast	6 (29%)	20 (30%)
Colorectal	0 (0%)	4 (6%)
Hematological	1 (5%)	6 (9%)
Lung	2 (10%)	9 (14%)
Prostate	7 (32%)	15 (23%)
Other	5 (24%)	12 (18%)
Trial		
Ketamine Pain Study	7 (33%)	21 (32%)
Pregabalin Bone Trial	14 (67%)	45 (68%)
Trial site		
Edinburgh	9 (43%)	30 (45%)
Glasgow	12 (57%)	36 (55%)

**Table 2:** Examples of Positive and Negative Influences From a Trial on a Patient’s Well-Being

Positive Influences	Negative Influences
Pain reduction	Taking too many pills
Altruistic acts	Failure of the trial to reduce pain
Trial staff input	Difficult questions to answer
The trial structure	Side effects

## Appendix I

### Clinical Trials

Pregabalin Bone Trial—this was a randomized, double-blind trial of pregabalin vs. placebo in patients with cancer-induced bone pain receiving palliative radiotherapy. After randomization, patients were allocated to pregabalin or placebo for a period of 28 days. During this time, pregabalin/placebo was titrated to effect when either pain improved or the maximum dose was reached.

Ketamine Pain Study—this was a randomized, double-blind trial of ketamine vs. placebo in patients with cancer-related neuropathic pain. After randomization, patients were allocated to ketamine or placebo for a period of 14 days. During this time, ketamine/placebo was titrated to effect when either pain improved or the maximum dose was reached. In patients whose pain improved, they continued in the study for a further 16 days (30 days in total).

## Appendix II

### First Interview Schedule

#### Reasons for Participating

Can you tell me how you came to hear about the trial?

What was your initial reaction to the trial and how did you come to be involved?

What were your reasons for agreeing to take part in the trial?

##### Areas that could be explored

Do you remember what your frame of mind was at the time of starting the trial (including mood and emotion)?

How were you coping with your acceptance of your disease at the time?

Was there any motivation to help others in taking part?

Did you feel that taking part would help improve your symptoms?

Could you tell me if taking part in the study affected your levels of hope?

#### Experience of the Trial

What were your experiences of the trial?

How well did you understand what you were participating in?

Do you feel you were fully informed of the requirements of the trial?

Had you been involved in previous research/trials?

Did you have any negative experiences during the trial?

##### Areas that could be explored

What were your experiences with the research staff (i.e., doctors and nurses)?

What impact did the trial have on your life?

### Last Interview Schedule

What do you remember about the trial?

Did being on the trial change how you see yourself as a person?

How do you look back on the period of time when you were on the trial?

What happened after the trial finished?

How did you find the change after the contact with the trial staff ceased?

If no benefit, has the trial been a waste of your time?

Can you remember anything that occurred that you were not expecting during the trial?

What area of the clinical trial could be better for patients like you?
